# Aerobic catabolism and respiratory lactate bypass in Ndh-negative *Zymomonas mobilis*

**DOI:** 10.1016/j.mec.2018.e00081

**Published:** 2018-11-15

**Authors:** Inese Strazdina, Elina Balodite, Zane Lasa, Reinis Rutkis, Nina Galinina, Uldis Kalnenieks

**Affiliations:** Institute of Microbiology and Biotechnology, University of Latvia, Jelgavas street 1, Riga LV-1004, Latvia

**Keywords:** Lactate dehydrogenase, Respiratory chain, NADH dehydrogenase, *Zymomonas mobilis*, Bioethanol, Oxidative stress

## Abstract

Ability to ferment in the presence of oxygen increases the robustness of bioprocesses and opens opportunity for novel industrial setups. The ethanologenic bacterium *Zymomonas mobilis* performs rapid and efficient anaerobic ethanol fermentation, yet its respiratory NADH dehydrogenase (Ndh)-deficient strain (*ndh-*) is known to produce ethanol with high yield also under oxic conditions. Compared to the wild type, it has a lower rate of oxygen consumption, and an increased expression of the respiratory lactate dehydrogenase (Ldh). Here we present a quantitative study of the product spectrum and carbon balance for aerobically growing *ndh-*. Ldh-deficient and Ldh-overexpressing *ndh-* strains were constructed and used to examine the putative role of the respiratory lactate bypass for aerobic growth and production. We show that aerobically growing *ndh-* strains perform fermentative metabolism with a near-maximum ethanol yield, irrespective of their Ldh expression background. Yet, Ldh activity strongly affects the aerobic product spectrum in glucose-consuming non-growing cells. Also, Ldh-deficiency hampers growth at elevated temperature (42 °C) and delays the restart of growth after 10–15 h of aerobic starvation.

## Highlights

• Aerobically growing Ndh-deficient *Z. mobilis* produce ethanol with maximum yield.
• Lactate bypass affects respiration and product yields in non-growing Ndh-deficient cells.
• Respiratory Ldh is involved in *Z. mobilis* oxidative and thermal stress resistance.

## 1 Introduction

An ideal producer microorganism must have an active product synthesis pathway with a high yield, ensuring that most of substrate carbon ends up in the target product. The bacterium *Zymomonas mobilis* serves as a classical example of this type of metabolism (Belaich and Senez, 1965). *Z. mobilis* possesses the most rapid ethanologenic pathway among microorganisms (Rogers et al., 1982), which is composed of the Entner–Doudoroff (E-D) glycolytic pathway together with pyruvate decarboxylase and alcohol dehydrogenase activities. Up to 98% of substrate carbon gets incorporated in ethanol. The outstanding ethanol-synthesizing ability of *Z. mobilis* has promoted metabolic engineering work on bioethanol production from renewable substrates (Zhang et al., 1995, Rogers et al., 2007, He et al., 2014, Chou et al., 2015), recently including genomic and synthetic biology perspective (Wang et al., 2018), as well as numerous attempts to increase its robustness to medium constituents and environmental conditions, like inhibitory compounds, high temperature, or presence of oxygen (Hayashi et al., 2012, Yang et al., 2014, Tan et al., 2016, Charoensuk et al., 2017).

Ability to produce ethanol or other anaerobic fermentation products with high yields in the presence of oxygen is a highly desirable property of a producer microorganism. Unhampered biomass growth with a fermentative type of metabolism in the presence of oxygen might open novel opportunities for industrial settings (Bennett and San, 2017, Wu et al., 2015). Apart from making fermentations more robust to air, that potentially enables co-cultivations with obligate aerobes, and perhaps, also with phototrophs, which evolve oxygen into the growth medium. High aerobic fermentation rates under non- or slowly-growing conditions adds further value, improving the yield of target product at the expense of biomass formation (Michalowski et al., 2017), and broadening potential applications of immobilized cells.

*Z. mobilis* seems to be fairly close to meet the above requirements. Catabolism in *Z. mobilis* is largely uncoupled from its anabolism, and proceeds with a high rate under non- or slowly-growing conditions (Belaich and Senez, 1965). Furthermore, recent evidence shows that respiratory mutants of *Z. mobilis* are able to grow and perform fermentative type of metabolism in the presence of oxygen (Kalnenieks et al., 2008, Hayashi et al., 2011). *Z. mobilis* bears an active respiratory chain, with the type II NADH dehydrogenase (Ndh), coenzyme Q_10_, and the cytochrome *bd* terminal oxidase as the established major electron carriers, together with D-lactate dehydrogenase, cytochrome *c* peroxidase and some other minor or still unidentified constituents (Balodite et al., 2014, Charoensuk et al., 2011, Kalnenieks et al., 1998, Sootsuwan et al., 2008, Strohdeicher et al., 1990). For some reason, the energetic efficiency of *Zymomonas* respiration is lower than reported for other bacteria with similar composition of electron transport chain (Rutkis et al., 2014), which makes it quite unique among the producer microorganisms. *Z. mobilis* does not use its respiration to supply energy for aerobic growth in the same way as the majority of aerobic and facultatively anaerobic microorganisms do. In contrast to such prominent facultatively anaerobic “workhorses” of biotechnology as *E. coli* and *B. subtilis*, and even to *Lactococcus lactis* (which bears an inducible electron transport chain with low coupling efficiency, resembling the major electron pathway in *Z. mobilis*; Brooijmans et al., 2007), aeration does not improve *Zymomonas* biomass yield (Belaich and Senez, 1965, Kalnenieks, 2006). Yet, it lowers the ethanol yield, since respiration withdraws the reducing equivalents from the alcohol dehydrogenase reaction and causes accumulation of the inhibitory metabolic precursor of ethanol, acetaldehyde. Due to accumulation of acetaldehyde, aerobic conditions tend to inhibit *Z. mobilis* growth. However, *Z. mobilis* in the presence of respiratory inhibitors (cyanide), and more specifically, its Ndh-deficient mutant strains under aerobic conditions grow better than the wild type. They produce more ethanol, without accumulation of acetaldehyde (Kalnenieks et al., 2000, Kalnenieks et al., 2008, Hayashi et al., 2011, Hayashi et al., 2012).

Although in general the Ndh-deficient *Z. mobilis* looks promising for aerobic bioprocess applications, our basic understanding of its catabolism is insufficient. Quantitative data on its aerobic product spectrum and yield values are lacking. Furthermore, our previous study indicated that inactivation of *ndh* elicited certain adaptive response at the level of the electron transport chain and oxidative stress protection (Strazdina et al., 2012). In particular, the Ndh-deficient *Z. mobilis* strain showed increased activity of the respiratory D-lactate oxidase (Ldh). This finding posed further questions, concerning the general relation of Ldh activity to the aerobic physiology of the mutant – its growth, aerobic persistence, and metabolism. *Z. mobilis* possesses two distinct enzymatic activities of lactate dehydrogenase: the cytoplasmic NAD^+^-dependent lactate dehydrogenase (Lawford and Rousseau, 2002) and the membrane-bound FAD-dependent respiratory lactate dehydrogenase (Kalnenieks et al., 1998). In the Ndh-deficient phenotype, these lactate dehydrogenase enzymes may function in tandem, forming the so called respiratory lactate bypass. The cytoplasmic NAD^+^-dependent enzyme produces lactate by reducing pyruvate, while the respiratory lactate dehydrogenase reoxidizes lactate, regenerating pyruvate. The net result of this shunt is oxidizing of NADH and transferring reducing equivalents to the respiratory chain. Lactate bypass has been shown to operate in an NADH dehydrogenase - deficient strain of the bacterium *Corynebacterium glutamicum* (Nantapong et al., 2004), where its supposed function is to fuel the electron transport chain with electrons, and hence, to maintain the respiratory ATP yields comparable to those of the wild type.

In the present work our aim was to carry out a quantitative study of the product spectrum and carbon balance in aerobically growing *Z. mobilis* with an Ndh-deficient background, as well as to examine the putative role of the respiratory lactate bypass for growth and production in these strains. In our previous study and in the works of Hayashi et al., 2011, Hayashi et al., 2012, *Z. mobilis* ZM6 (ATCC 29191) was chosen for construction of Ndh-deficient mutants, so here we used the existing ZM6 Ndh-deficient strain as the background for further Ldh mutant derivatives. We show that aerobically growing Ndh-deficient strains, whether bearing an Ldh-deficient or Ldh-overexpressing background, equally well perform fermentative metabolism with a near-maximum ethanol yield. Yet, Ldh strongly affects the aerobic product spectrum in glucose-consuming non-growing cells, and also, its deficiency hampers growth at elevated temperature and delays restart of growth after aerobic starvation.

## 2 Material and methods

### 2.1 Bacterial strains, plasmids and transformation

*Z. mobilis* strains ATCC 29191 (Zm6) and its Type II respiratory NADH dehydrogenase (Ndh)-deficient mutant derivative were cultivated and maintained as described previously (Kalnenieks et al., 1993, Kalnenieks et al., 2008, Strazdina et al., 2012). Strain *Escherichia coli* JM109 was used as the host for cloning of the recombinant plasmids. *E. coli* JM109 and plasmid pGEM-3Zf (+) were purchased from Promega. The plasmids and strains constructed and studied in the present work are listed in Table 1. *Z. mobilis* were transformed by electroporation (Zou et al., 2012). *E. coli* was transformed by calcium chloride assay as described by Sambrook et al. (1989).

**Table 1:** Plasmids and strains used in the study.

**Plasmids**	**Characteristics**	**Source**
pBBR1MCS-2	Plasmid vector; kan^r^	Addgene
pGEM-3Zf(+)	Plasmid vector; amp^r^	Promega
pBR322	Plasmid vector; tet^r^	Promega
pGEMldh	pGEM-3Zf(+) derivative, carrying a 1.32 kb fragment of PCR-amplified genomic DNA, representing the major part of the ORF of respiratory D-lactate dehydrogenase (*ldh*; ZZ6 RS04930), cloned between *Bam*HI and *Hind*III sites of the MCS	Present work
pGEMldh::tet^r^	pGEMldh derivative, carrying a 1.49 kb fragment of PCR-amplified DNA, corresponding to the tetracycline resistance determinant of pBR322, inserted in the *Age*I site of *ldh*	Present work
pBBRgap	pBBR1MCS-2, containing an 0.2 kb fragment of the chromosomal DNA, corresponding to the promoter region of the glyceraldehyde-3-phosphate dehydrogenase gene (ZZ6 RS05365), cloned between the *Sac*I and *Bam*HI sites of the MCS	Present work
pBBRgapldh	pBBRgap derivative, carrying a 1.73 kb fragment of PCR-amplified genomic DNA with the ORF of *ldh*, cloned between *Bam*HI and *Hind*III sites of the MCS, next to the gap promoter	Present work
**Strains**		
*ndh-*	Zm6 strain with a cm^**r**^ insert in the ORF of respiratory type II NADH dehydrogenase gene (*ndh*)	Kalnenieks et al. (2008)
*ndh-ldh-*	*ndh-* strain with a tet^**r**^ insert in the ORF of respiratory D-lactate dehydrogenase gene (*ldh*)	Present work
*ndh-ldh+*	*ndh-* strain, transformed with pBBRgapldh	Present work
*ndh-ldh-ldh+*	*ndh-ldh-* strain, transformed with pBBRgapldh	Present work

### 2.2 Cloning techniques and strain construction

Isolation of genomic and plasmid DNA from *Z. mobilis* was performed as described before (Kalnenieks et al., 2008, Strazdina et al., 2012). A 1.32 kb region of *Z. mobilis* chromosomal DNA, including the major part of the ORF of respiratory D-lactate dehydrogenase gene (*ldh*; *Z. mobilis* Zm6 genome sequence, locus tag ZZ6_RS04930) was amplified by PCR, using the primer pair Dlactdeh1 and Dlactdeh2 (Table 2). For construction of *ldh* knock-out mutant, the amplified DNA fragment was double-digested with *Bam*HI and *Hin*dIII, and directionally cloned between the *Bam*HI and *Hin*dIII restriction sites of the multiple cloning site (MCS) of plasmid pGEM-3Zf(+), yielding plasmid pGEMldh (Table 1). pGEMldh was used to transform *E. coli* JM109, and the transformants were plated on LB agar with ampicillin (100 μg mL^−1^). The cloned *ldh* fragment contained an intrinsic *Age*I restriction site, allowing to cut it in two pieces of 0.27 kb and 1.05 kb length. This restriction site was used for insertional inactivation of the gene. Tetracycline resistance determinant (tet^r^) was amplified by PCR, with primers TetAge1 and TetAge2, taking the tet^r^-containing plasmid pBR322 as the template. The amplified 1.49 kb DNA fragment was digested with *Age*I, and inserted into the *Age*I site of the cloned *ldh*. The resulting plasmid construct pGEMldh::tet^r^ (Table 1) was used to transform *E. coli* JM109. Transformants were plated on LB agar with ampicillin (100 μg mL^−1^) and tetracycline (20 μg mL^−1^). This plasmid construct, unable to propagate in *Z. mobilis*, was then used to transform the *Z. mobilis ndh*-deficient mutant (strain *ndh-*, carrying chloramphenicol resistance insert in *ndh*), and colonies of homologous recombinants (the genotype *ndh-ldh-*; Table 1) were selected on plates containing chloramphenicol (120 μg mL^−1^) and tetracycline (20 μg mL^−1^). As expected, PCR reaction on the chromosomal DNA from these colonies with the primer pair TetAge1 and Dlactdeh1, yielded a product of 1.7 kb length, corresponding to the whole of tet^r^ determinant along with an 0.27 kb fragment of the *ldh* ORF. Sequencing of the PCR product confirmed presence of tet^r^ in the *Age*I site of the chromosomal copy of *ldh*.

**Table 2:** PCR primers used in the study.

**Primer**	**Sequence**	**Restriction site (underlined)**
Dlactdeh1	CAGCAATTTCTGGATCCTCTTCGTG	*Bam*HI
Dlactdeh2	CAACGCAACGAAAGCTTCGACTTCATCC	*Hin*dIII
TetAge1	CAGCTTATCACCGGTAAGCTTTAATGCGG	*Age*I
TetAge2	GATTCATTCTGCTAACCGGTAAGGCAACCC	*Age*I
gap1	GACAATGAGCTCGGAACGGTATACTG	*Sac*I
gap2	CAACTTTAACCGCCATGGATCCTCTC	*Bam*HI
memldh1	GGTTGGATCCATGGTGCAGCTTCC	*Bam*HI
memldh2	GCAAAGCTTCTATCTCCAATAAGC	*Hin*dIII

The plasmid construct used for complementation of the *ldh* knockout mutation, and for overexpression of the *ldh* gene, was built on the basis of the shuttle vector pBBR1MCS-2, containing kanamycin resistance marker. First, an 0.2 kb fragment of the chromosomal DNA, corresponding to the promoter region of the glyceraldehyde-3-phosphate dehydrogenase gene, Pgap (*Z. mobilis* Zm6 genome sequence, locus tag ZZ6_RS05365) was amplified with the primer pair gap1 and gap2. The amplified fragment was double-digested with *Sac*I and *Bam*HI, and directionally cloned between the respective restriction sites of the MCS of shuttle vector pBBR1MCS-2, yielding plasmid pBBRgap (Table 1). After that, a 1.73 kb DNA fragment, including the whole ORF of *ldh*, was amplified on the chromosomal DNA template with the primers memldh1 and memldh2, double-digested with *Bam*HI and *Hin*dIII, and directionally cloned between the respective sites in pBBRgap (positioning the *ldh* ORF next to the cloned Pgap promoter region). The resulting construct pBBRgapldh (Table 1) was cloned in *E. coli* JM109, verified by sequencing, and used to transform *Z. mobilis* both for Ldh overexpression and for complementation of the *ldh* gene knockout. To select the strain *ndh-ldh+* , overexpressing Ldh against the background of the *Z. mobilis ndh-*, transformants were plated on agar plates with chloramphenicol (120 μg mL^−1^) and kanamycin (310 μg mL^−1^). For complementation of the strain ndh-ldh-, transformants were selected on plates containing chloramphenicol, tetracycline and kanamycin, yielding *Z. mobilis* strain *ndh-ldh-ldh+* .

Reactants, equipment and assays for PCR, as well as the RT-qPCR assay and the corresponding primers for quantification of *ldh* transcripts, were the same as used by Strazdina et al. (2012). Other DNA manipulations and verification of DNA constructs by sequencing were carried out as described previously (Kalnenieks et al., 2008, Strazdina et al., 2012).

### 2.3 Cultivation of strains and experiments with non-growing cell suspensions

Inocula for cultivation experiments were grown overnight in 30 mL Falcon tubes without shaking in a thermostat at 30 °C on standard growth medium with 5 g L^−1^ yeast extract, 50 g L^−1^ glucose and mineral salts (Kalnenieks et al., 1993), with strain-specific selective antibiotics added (see above). Cultivation experiments for all strains also were carried out at 30 °C (if not stated otherwise) in the same growth medium, but without addition of antibiotics. It was shown previously that the recombinant strains, transformed with our pBBR-based plasmid constructs, retained their stability without selective pressure for at least 50 generations.

Aerobic cultivations were done in batch mode, in 200 mL shaken flasks with 20 mL culture at 180 r.p.m. Anaerobic cultivations were done in 18 mL glass screw cap tubes containing 10 mL of culture. The tubes were vigorously gassed with nitrogen for a period of several minutes after inoculation, as well as regularly during the sampling.

For aerobic experiments with non-growing cell suspensions, cells were harvested from overnight cultures, washed and resuspended in 150 mM potassium phosphate buffer, pH 6, supplemented with 5 mM magnesium sulfate and 200 g L^−1^ glucose. Suspensions were incubated under three aeration modes: (i) in 200 mL shaken flasks with 20 mL of cell suspension at 180 r.p.m., (ii) in a 0.5 L tabletop fermenter Sartorius Stedim Biotech, model Biostat Q Plus, containing 150 mL of cell suspension, mixed at 200 r.p.m. and gassed with air at flow rate of 1 vol (vol of cell suspension)^−1^ min^−1^, and (iii) in 18 mL glass screw cap tubes, containing 10 mL of cell suspension, gassed during the experiment with air at flow rate of 5 vol (vol of cell suspension)^−1^ min^−1^.

For aerobic starvation experiments, cells were harvested from overnight cultures, grown in thermostat without shaking on growth medium with 20 g L^−1^ glucose, washed and resuspended in 100 mM potassium phosphate buffer, pH 7, at biomass concentration of 1.0–1.2 mg dry wt (mL)^−1^. Aliquots of 3 mL were transferred into 30 mL Falcon tubes, and incubated on a rotary shaker at 120 r.p.m. for distinct time spans. After that, 20 microliter aliquots of starved cell suspensions were inoculated in wells of a 96-well plate, each containing 180 microliters of fresh medium of the same composition, and cultivated without aeration in a Tecan plate reader.

### 2.4 Preparation of cell-free extracts and membranes

For preparation of cell-free extracts, cells were harvested by centrifugation at 6000 *g* for 10 min. After sedimentation, approx. 1 mL of the wet pellet was transferred into the vessel of Retsch MM301 disintegrator, glass beads (106 µm average diameter) were added, and 2 times of 1.5 min vortexing at 30 Hz frequency was performed with 1 min interval for cooling. After that, unbroken cells together with glass beads were sedimented by centrifugation at the same regime. Typically, following this procedure cell-free extract with protein concentration around 2 mg mL^−1^ was obtained. Cytoplasmic membrane preparations were obtained by ultracentrifugation of the cell-free extracts, as described previously (Kalnenieks et al., 1993, Kalnenieks et al., 1998).

### 2.5 Enzymatic assays

Lactate oxidase assay contained 500 μL of the cell-free extract with protein concentration around 2 mg mL^−1^ and 480 μL of 100 mM potassium phosphate buffer, pH 7 (or alternatively, 30–50 μL of membrane preparation with protein concentration of 5–6 mg protein mL^−1^ and 950–930 μL of buffer, respectively). The reaction was started by addition of 20 μL of 2 M D-lactate, and oxygen uptake was measured with Clark electrode. Lactate dehydrogenase in membranes was monitored by measuring lactate:CoQ_1_ oxidoreductase activity, using oxidised coenzyme Q_1_ instead of oxygen as the terminal electron acceptor. 1 mL of assay in a spectrophotometer cuvette contained 100 mM potassium phosphate buffer, 30 μL of membranes, 10 μL of 8 mM CoQ_1_ and 20 μL of 1 M potassium cyanide. The reaction was started by adding 20 μL of 2 M D-lactate, and the absorbtion decrease at 276 nm wavelength was followed spectrophotometrically. For calculations, 14.3 mM^−1^cm^−1^ was taken for the millimolar extinction coefficient of oxidised CoQ_1_. The NADH dehydrogenase (NADH: CoQ_1_ oxidoreductase) assay was similar, except that it contained 10 μL membranes, and was started by addition of 20 μL 10 mM NADH instead of D-lactate. The reaction was followed by monitoring absorbtion change at 340 nm, taking 6.22 mM^−1^cm^−1^ as the millimolar extinction of NADH. Alcohol dehydrogenase and pyruvate decarboxylase activities were measured as described in Rutkis et al. (2016).

### 2.6 Analytical methods

Glucose, ethanol, lactate, acetate and glycerol concentrations were determined by HPLC (Agilent 1100 Series), using column Shodex (length 300 mm, internal diameter 8 mm) with a refractive index detector. The column temperature was 50 °C, with 5 mM M H_2_SO_4_ as the mobile phase at flow rate of 0.6 mL min^−1^, and the sample volume was 5 μL. Concentration of dissolved oxygen was monitored by Clark-type Thermo Scientific Orion dissolved oxygen probe, assuming oxygen concentration of air-saturated buffer solution to be 0.24 mM (Truesdale et al., 1955). Protein concentration in cell-free extracts and membrane samples was determined according to Markwell et al. (1978). Cell concentration was determined as OD_550_, and dry cell mass of the suspensions was calculated by reference to a calibration curve. Results are means of three replicates, if not stated otherwise. Standard deviation values are given as error bars in the figures.

## 3 Results

### 3.1 Respiratory lactate dehydrogenase and lactate oxidase activity

To confirm the mutant phenotypes, respiratory D-lactate oxidase and D-lactate:coenzyme Q_1_ oxidoreductase activities were monitored in membrane vesicles and cell-free extracts, prepared from late exponential phase aerobically growing cultures. Results are presented in the Table 3. The *ldh* knock-out mutation led to a complete loss of lactate dehydrogenase activity. In the membranes of the strain *ndh-ldh-*, neither the lactate:CoQ_1_ oxidoreductase, nor lactate oxidase could be detected. On the other hand, lactate dehydrogenase (and oxidase) activity could be restored by transformation of *Z. mobilis* with the plasmid construct pBBRgapldh. Transformation with pBBRgapldh yielded expression of a functional lactate dehydrogenase. This was evident from the successful complementation of the *ldh* knock-out mutation, and also, from the elevation of activity in the strain *ndh-ldh+* approximately 1.5–2.5 times above the levels of *ndh-*. Variation of enzymatic activities basically corresponded to the RT-qPCR data on the relative *ldh* transcript levels; the mean log_2_ ratio values are presented in Table 3.

**Table 3:** Lactate:CoQ_1_ oxidoreductase and lactate oxidase activity, U (mg prot)^−1^, and relative transcript levels of *ldh* in the mutant strains.

Strain:	*ndh-*	*ndh-ldh+*	*ndh-ldh-*	*ndh-ldh-ldh+*
Lactate: CoQ_1_ oxidoreductase activity (in membranes)	0.039 ± 0.003	0.099 ± 0.006	0.000 ± 0.001	0.028 ± 0.002
Lactate oxidase activity (in cell-free extracts)	0.082 ± 0.020	0.112 ± 0.042	0.000 ± 0.004	0.068 ± 0.002
RT-qPCR log_2_<mutant/*ndh*->	–	3.3	− 8.1	− 0.4

### 3.2 Growth and stress tolerance of the mutant strains

**Fig. 1:**
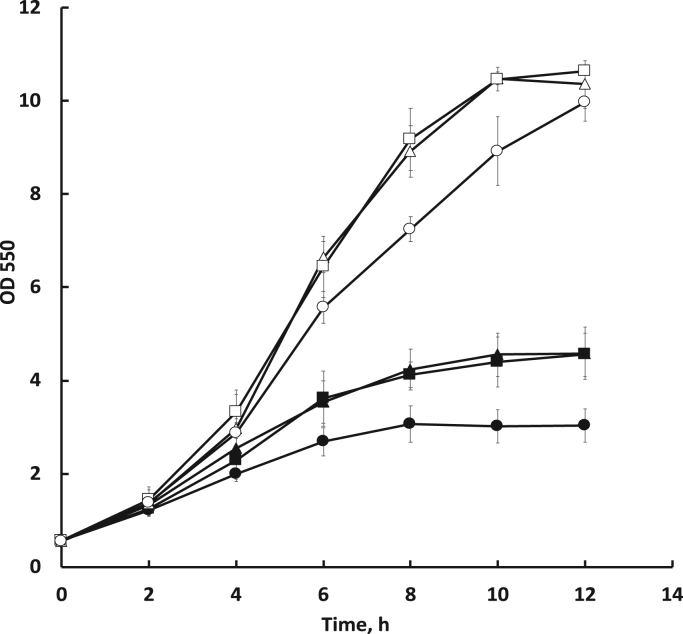
Batch growth under aerobic conditions in shaken flasks at 30 °C (open symbols) and 42 °C (filled symbols). Triangles – *ndh-*, squares – *ndh-ldh+*, circles – *ndh-ldh-*. Mean values of four cultivation experiments are shown.

Since previously *Z. mobilis* with Ndh-deficient phenotype was shown to have an improved aerobic growth and temperature tolerance (Kalnenieks et al., 2008, Hayashi et al., 2011), and at the same time, an elevated respiratory Ldh activity (Strazdina et al., 2012), we were interested to examine the stress tolerance of the newly obtained *ndh-* strains with different Ldh backgrounds. Aerobic batch cultivations at two temperatures are shown in the Fig. 1. At 30 °C the strain *ndh-ldh-* tended to grow with a slightly lower specific growth rate and biomass yield than the others. Yet, after 12 h of cultivation, as the early stationary phase was reached, biomass concentrations in all three cultures were similar.

Growth differences turned out to be more pronounced, when strains were either cultivated aerobically at 42 °C, or put to a short term aerobic starvation at 30 °C before cultivation. As seen in the Fig. 1, the growth rates of all strains at 42 °C were much lower than at 30 °C. However, the advantage of *ndh-* and *ndh-ldh+* over *ndh-ldh-* had become more substantial: after 12 h of growth they reached significantly higher biomass concentrations, than the double knock-out strain. The strains *ndh-* and *ndh-ldh+* grew with a closely similar kinetics at both temperatures, implying that the aerobic growth capacity and thermal tolerance of *ndh-* could hardly be improved by further overexpression of Ldh. On the other hand, in the Ldh-deficient background the stress tolerance was reduced.

**Fig. 2:**
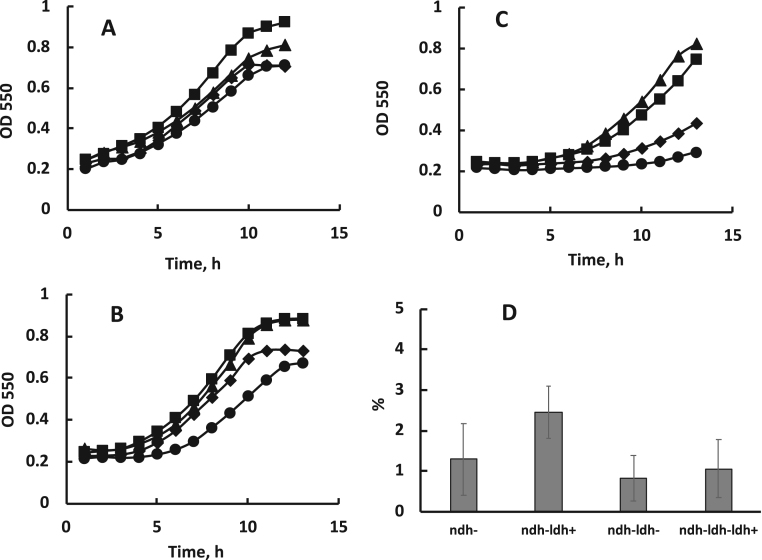
Batch growth under microaerobic conditions and relative decrease of viable cell numbers after aerobic starvation of cells in phosphate buffer in shaken flasks. Growth after (A) 6 h, (B) 10 h, (C) 15 h, shown as representative growth curves of one of four independent experiments in 96-well plates; triangles – *ndh-*, squares – *ndh-ldh+*, diamonds – *ndh-ldh-ldh+*, circles – *ndh-ldh-*. (D) cfu after 15 h of aerobic starvation relative to the respective values from cell suspensions at the start of starvation. No statistically significant differences between any two of the strains were found (P > 0.05). Viable cell counts represent means of 5 starvation experiments.

The time needed for strains to restart growth after 6, 10, or 15 h starvation at 30 °C also differed (Fig. 2). The 6 h period of starvation seemed to have little effect on any of the strains, yet 10 h, and especially, 15 h of aerobic starvation increased the duration of the lag phase, primarily in the strain *ndh-ldh-*. In the complemented strain *ndh-ldh-ldh+*, the length of lag phase was closer to that of strains *ndh-* and *ndh-ldh+*. It seemed plausible that extension of lag phase could be due to decreased viable cell numbers at the end of starvation period. Yet, although the aerobic starvation strongly reduced the amount of cfu in all four strains, the relative decrease of viable cell counts did not vary much between the strains and poorly correlated with the extension of lag phase. For *ndh-ldh-* the viable cell counts after 15 h starvation did not differ significantly from those of *ndh-* (Fig. 2D), while for *ndh-ldh+*, showing slightly higher viable cell numbers, the growth curve was very close to that of *ndh-*.

Data on the biomass yields and specific growth rates of a larger set of batch cultivation experiments is presented in the Supplement Table 1. Growth of the mutant strains was examined under aerobic and anaerobic conditions at 30 °C, in standard media with 5% glucose, both with or without 30 mM D-lactate supplement. Lactate was added as an external substrate of the respiratory Ldh, aiming to make the putative physiological effects of lactate-dependent respiration more explicit. It appeared however, that addition of lactate did not affect the growth rate, and neither did it raise the aerobic biomass yield relative to its anaerobic value in any of the strains. Irrespective of aeration, the strain *ndh-ldh-* tended to grow with a slightly lower specific growth rate and biomass yield than the others. For all strains the specific growth rate in batch cultures under anaerobic conditions exceeded the aerobic values.

### 3.3 Lactate metabolism and the aerobic fermentation balance

**Fig. 3:**
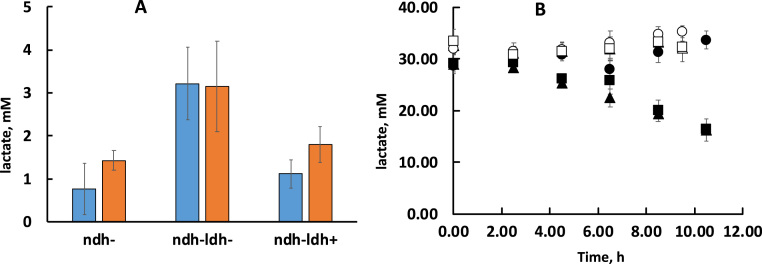
Lactate production (A) and consumption of exogenously added lactate (B) by the mutant strains during aerobic and anaerobic batch growth. In (A), lactate accumulation after 10 h of batch cultivation is presented. Blue bars, aerobic growth; orange bars, anaerobic growth. Differences between the lactate level in *ndh-ldh-* and those in both other strains were statistically significant (P < 0.001), and so were the differences between the aerobic and anaerobic condition in *ndh-ldh+* and in *ndh-* (P < 0.05), but not in *ndh-ldh-*. In (B) triangles refer to *ndh-*; circles – *ndh-ldh-*; squares – *ndh-ldh+*; filled symbols, aerobic growth; empty symbols, anaerobic growth. Mean values of 3 experiments are shown.

Analysis of fermentation products revealed differences in lactate metabolism between the strains. Apart from the major fermentation product ethanol, some minor accumulation of lactate took place, reaching millimolar concentration range in all three strains by the beginning of stationary phase (Fig. 3A). In the strain *ndh-ldh-*, lactate concentration reached the highest value, around 3 mM, and its accumulation did not depend on aeration. Notably, in strains *ndh-* and *ndh-ldh+* lactate accumulation did depend on aeration: relatively less lactate was accumulated in aerated cultures. Under anaerobic conditions the lactate concentration in these strains was higher, although not reaching the level seen in *ndh-ldh-*.

Dependence of lactate accumulation on the culture aeration suggested that endogenously generated lactate might be continuously reoxidised by the respiratory lactate oxidase throughout the aerobic cultivation. For further support of the function of respiratory Ldh in aerated culture, its activity was monitored also in the cultures with exogenously added lactate. In a series of cultivations, where the growth medium was supplemented with lactate (Fig. 3B), no net uptake of lactate was seen in *ndh-ldh-*, either aerobically or anaerobically. The strains *ndh-* and *ndh-ldh+* slowly consumed the added lactate, albeit only under aerobic conditions.

Table 4 shows the carbon balance for aerobic batch cultures without external D-lactate addition. Carbon recovery was very close to 100% for all three mutant strains, with ethanol being the major product. For calculations we assumed equimolar production of CO_2_ and ethanol, and CO_2_ and acetate (CO_2_ evolution was not measured directly), and 24.625 g were taken for dry weight of C-1 mol of biomass (Kremer et al., 2015). It appeared that ethanol was produced with a near-maximum yield (around 1.9 mol per mol of glucose; Table 4). Note that in the actively respiring wild type strain Zm6 ethanol yield under similar culture conditions reached merely half of its maximum value (Kalnenieks et al., 2008). This result indicated that in aerobically growing mutant strains catabolism was strictly ethanologenic, with almost no respiratory component and minor byproduct synthesis.

**Table 4:** Biomass and product yields in aerobic batch cultures. Results represent mean of three cultivation experiments in shaken flasks. Yield values refer to 10 h of cultivation (early stationary phase).

Strain	Yx/glucose [g dry wt mol^−1^]	YetOH/glucose [mol mol^−1^]	Ylactate/glucose [mol mol^−1^]	Yglycerol/glucose [mol mol^−1^]	Yacetate/glucose [mol mol^−1^]	C recovery [%]
*ndh-*	7.773 ± 0.212	1.931 ± 0.052	0.002 ± 0.003	0.005 ± 0.002	0.020 ± 0.007	103.1
*ndh-ldh-*	7.090 ± 0.360	1.891 ± 0.033	0.019 ± 0.007	0.004 ± 0.001	0.012 ± 0.003	101.1
*ndh-ldh+*	8.483 ± 0.653	1.913 ± 0.040	0.004 ± 0.004	0.005 ± 0.003	0.021 ± 0.004	102.9

Analysis of product yields was supported by direct measurement of culture respiration. Oxygen consumption was measured at regular time intervals in culture samples, which were rapidly transferred from the shaken flasks into the Clark electrode chamber, and their respiration rate monitored without external substrate addition. In all three strains during the exponential phase of growth oxygen was taken up at the specific rate around 0.01–0.02 U mg dry wt^−1^ (not shown), which was more than an order of magnitude below the values previously reported for aerobically growing Zm6 culture under similar conditions (Kalnenieks et al., 2000).

### 3.4 Aerobic catabolism in non-growing cells

Apart from growing cultures, glucose fermentation was monitored also in washed cell suspensions. That was done to examine the capacity of the engineered strains to perform aerobic bioconversion of high concentration sugary substrates using non-growing cells as biocatalysts. For these experiments we chose the extremes of the putative lactate bypass, with either none (*ndh-ldh-*), or with overexpressed (*ndh-ldh+*) Ldh activity. Cells were harvested from overnight cultures, washed and resuspended at 4 mg dry wt (mL)^−1^ in potassium phosphate buffer with glucose at 200 g L^−1^ final concentration, and incubated under three different modes of aeration (see Material and methods): in shaken flasks, fermenter (with 1 vol vol min^−1^ air flow), and glass screw cap tubes (with 5 vol vol min^−1^ air flow).

Like for growing cultures, samples of glycolysing cell suspensions from the shaken flasks at regular time intervals were transferred into Clark electrode chamber, and their respiration monitored without extra substrate addition. In contrast to aerobically growing cultures, respiration rates between the non-growing cells of *ndh-ldh-* and *ndh-ldh+* differed significantly. While for *ndh-ldh-* it was 0.012 U mg dry wt^−1^ (± 0.001), remarkably close to the values seen in growing culture, for the non-growing glycolysing cells of *ndh-ldh+* the respiration rate had more than tripled, and reached 0039 U mg dry wt^−1^ (± 0.006).

**Fig. 4:**
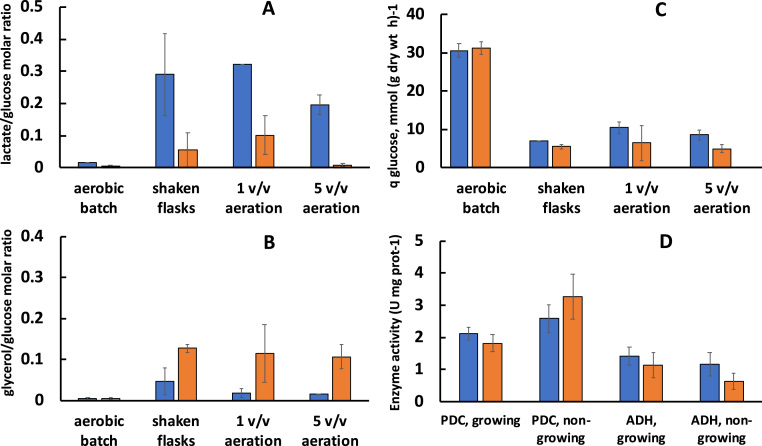
Non-volatile reduced byproduct yields (A, B), specific rate of glucose consumption (C), and activity of ethanologenic enzymes (D) in aerobically growing and non-growing cells. Pyruvate decarboxylase (PDC) activity and total alcohol dehydrogenase (ADH) activity was monitored in cell-free extracts, prepared after 8 h of aerobic growth, or incubation of non-growing cells, in shaken flasks. Blue bars: *ndh-ldh-*, orange bars: *ndh-ldh+* .

Accordingly, non-growing cells differed also with respect to their byproduct synthesis and glucose consumption rate (Fig. 4). In these experiments we focussed on non-volatile, reduced byproducts, lactate and glycerol, willing to monitor the redistribution of reducing equivalents among alternative routes, apart from those of ethanol synthesis and the respiratory chain. Under conditions of vigorous gassing we did not aim to reach a complete product balance (one, including ethanol, volatiles like acetate, acetaldehyde, and other minor byproducts). The strain *ndh-ldh-* in all three experimental setups showed high molar yield of lactate (Fig. 4A). In aerated fermenter, lactate yield for *ndh-ldh-* exceeded 0.3 mol per mole of consumed glucose. The strain *ndh-ldh+* yielded much less lactate. Yet instead, *ndh-ldh+* accumulated glycerol, with a stoichiometry of more than 0.1 mol per mole of glucose (Fig. 4B). The specific rate of glucose consumption in non-growing cells of both strains was lower than in the respective aerobic batch cultures (Fig. 4C). The non-growing cells of *ndh-ldh+* had significantly lower glucose uptake rate, than cells of *ndh-ldh-*. It is important to note that the byproduct yield values for non-growing cells by far exceeded those of the respective shaken flask cultures (compare ‘aerobic batch’ on the left side of Fig. 4A and B). On the other hand, ethanol yield in non-growing cell suspensions was lower than in the growing cultures. In the shaken flask incubations, ethanol to glucose molar ratio was 1.50 for strain *ndh-ldh-*, and only 1.26 for strain *ndh-ldh+* (not shown). For other experimental setups the ethanol yield was even lower. This could be due to production of lactate, glycerol, as well as volatiles, which evaporated under vigorous gassing. In both strains incubation under non-growing conditions lead to some increase of pyruvate decarboxylase (PDC) and a decrease of alcohol dehydrogenase activity (ADH) (Fig. 4D). While in *ndh-ldh-* that was seen as a slight tendency (although not meeting strict criteria of statistical significance), in *ndh-ldh+* the rise of PDC (P < 0.01) and decrease of ADH (P < 0.05) activity under non-growing conditions was more pronounced. The decrease of ADH activity apparently contributed to the observed shift from ethanol synthesis to other reduced byproduct accumulation.

## 4 Discussion

Our present results show that *Z. mobilis* strains with Ndh-deficient phenotype are very efficient at producing ethanol under aerobic culture conditions. Overexpression or inactivation of the membrane respiratory lactate dehydrogenase did not affect the ethanol yield, which was close to its theoretical maximum value in all aerobically growing Ndh-deficient strains. Also, the specific rate of glucose conversion was high. In aerobically growing mutants the specific rate of glucose consumption reached 30 mmol (5.4 g) g dry wt h^−1^. This value three times exceeds the aerobic glucose consumption rate recently reported for *E. coli* strain engineered for high glucose throughput (Michalowski et al., 2017). The Ndh-deficient strains can serve to carry out fermentative catabolism in various syntrophic bioprocess settings, where aerobic conditions are essential. Those might be syntrophies with oxygen-evolving photosynthetic microorganisms, resembling the recently described mixed culture of cyanobacterium *Synechococcus elongatus*, phototrophically overproducing glucose, with *Pseudomonas putida* utilizing glucose and accumulating polyhydroxyalkanoates (LÖwe et al., 2017), or alternatively, co-cultures between ethanol-producing *Z. mobilis* and microorganisms, which utilize ethanol as a substrate for aerobic biosynthesis (e.g., for accumulation of polyunsaturated fatty acids). Furthermore, ethanol is not the only product of interest. Ndh-deficient background in future may be exploited for construction of recombinant strains, producing other reduced catabolic products, like 2,3-butanediol (Yang et al., 2016), while growing under aerobic syntrophic settings.

Here we demonstrate that the respiratory lactate bypass is operative in aerobically growing Ndh-deficient *Z. mobilis*, albeit it functions at a low rate. Obviously, in *Zymomonas* it is not generating energy via oxidative phosphorylation for supporting growth. Its operation in growing cultures is slow, presumably because of the active pyruvate decarboxylase (PDC) and alcohol dehydrogenase (ADH), which scavenges most of pyruvate generated by the E-D pathway, and catalyzes its decarboxylation and conversion to ethanol. In result, the supply of reducing equivalents to the respiratory chain via the lactate bypass is very limited, with a negligible effect upon the ethanol yield. At the same time, the Ldh-deficient strain shows distinct signs of impaired growth. Those are: slightly lower specific growth rate, lower biomass concentration reached by the end of batch growth, and lower biomass yield values. The growth impairment caused by Ldh-deficiency becomes more vivid under stress conditions.

Previously it has been reported that the Ndh-deficient strains of *Z. mobilis* have growth advantage over the parent strain at elevated temperature (Hayashi et al., 2011, Hayashi et al., 2012) and also under aerobic conditions (Kalnenieks et al., 2008, Hayashi et al., 2011). It was thought to be somehow linked to their low respiratory rates and to the fact that they produce smaller amounts of the inhibitory metabolite acetaldehyde. Mechanistic details behind the respiration deficiency-caused thermotolerance so far remain obscure. Our present results, however, demonstrate that low respiration rate and Ndh-deficiency per se is not improving *Zymomonas* thermotolerance. The double mutant *ndh-ldh-*, which is lacking its Ndh activity, and having a near-zero respiration rate, has the thermosensitive phenotype of the parent strain Zm6. Perhaps, some threshold level of the respiratory Ldh activity is critical for thermotolerance, and this is why strains *ndh-* and *ndh-ldh+* are more thermotolerant than *ndh-ldh-* and Zm6. Ldh is involved also in maintaining the cell ability to restart growth after aerobic starvation. After 10–15 h of aerobic starvation at 30 °C the strain *ndh-ldh-* shows a substantially longer lag phase than the strains *ndh-* and *ndh-ldh+* , although the viable counts for all strains are comparable.

At least two mechanisms of the stress-protective action of Ldh can be put forward as working hypotheses. First, it is known that the thermal tolerance of bacteria under aerobic conditions largely depends on their capacity to deal with the reactive oxygen species (ROS). There is more generation of ROS and more oxidative stress in the microbial cell as the temperature rises (Davidson and Schiestl, 2001). Theoretically, oxidative and thermal stress tolerance may be affected by minor electron transport branches with low flux rate, having limited direct effect on the oxidative ATP generation and catabolic product spectrum, but nevertheless participating in generation or elimination of ROS. Ldh may well represent an electron transport branch, involved in ROS elimination (e.g., transferring electrons from lactate to a respiratory peroxidase). Notably, Hayashi et al. (2011) demonstrated significantly reduced levels of intracellular hydrogen peroxide in the Ndh-deficient strains, and suggested that under high temperature condition they suffer lower oxidative stress, than the wild type. Whether this reduction of intracellular H_2_O_2_ concentration is due to the elevated Ldh activity in the Ndh-deficient background (relative to the wild type; see Kalnenieks et al., 2008) is yet to be established. Another role of Ldh might be related to regulation of the intracellular lactate concentration. Although lactate in aerobically growing cells is being produced at a low rate (and probably, this is even more true for starving cells), it might be gradually accumulating in the intracellular space and acidifying the cytoplasm, in case if its export from the cell represents a bottleneck. Under such conditions, the intracellular concentration of lactate would be determined by balancing the reactions of its synthesis and oxidation. Hence, the lactate bypass would be vital to maintain its concentration acceptably low. Further studies are in progress in our lab to verify these assumptions by monitoring intracellular concentration of lactate and ROS production in the mutant strains.

In glucose-consuming, non-growing cells lactate synthesis proceeds at a higher rate, than in growing cultures, manifesting itself either as lactate accumulation in the suspension of *ndh-ldh-*, or as an elevated oxygen consumption rate by the cells of *ndh-ldh+*, which reoxidise lactate via the respiratory lactate bypass. This implies more pyruvate to be redirected from pyruvate decarboxylase to the cytoplasmic NAD^+^-dependent lactate dehydrogenase. Evidence supporting such flux redistribution between pyruvate decarboxylase and cytoplasmic lactate dehydrogenase come from the first genome-scale transcriptomic and metabolomic study of *Z. mobilis* aerobic growth by Yang et al. (2009). They reported upregulation of cytoplasmic lactate dehydrogenase and downregulation of pyruvate decarboxylase and alcohol dehydrogenase (*adhB*) gene expression in aerobic stationary phase cells of the strain Zm4. They also noted that aerobic conditions elevated the intracellular pool of lactate and stimulated lactate accumulation in the medium. We speculate that in our experiments accumulation of lactate or glycerol in the aerated non-growing mutant cells served as additional metabolic sink for reducing equivalents under conditions of decreased ADH activity and low respiratory rate.

**Fig. 5:**
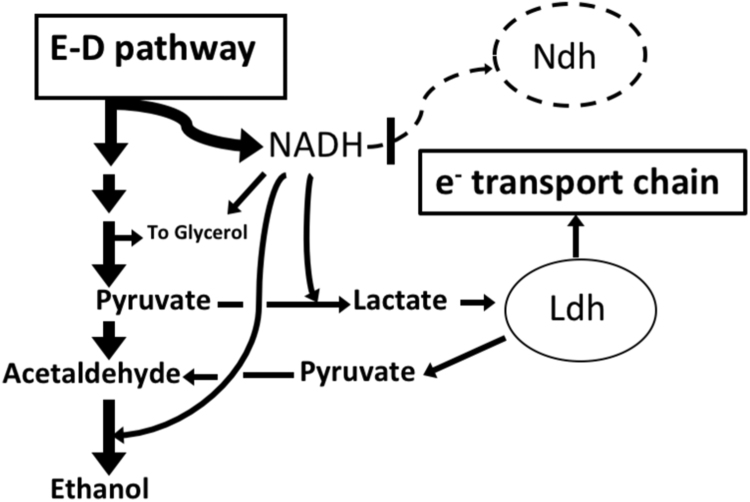
Redox balance and the respiratory lactate bypass in Ndh-deficient strains.

In summary (Fig. 5), in aerated Ndh-deficient cells the competition for reducing equivalents between the respiratory lactate bypass and ethanol synthesis determines the oxygen uptake rate, affects glucose consumption, and also shifts the catabolic product spectrum. Under certain bioprocess settings dependence of byproduct spectrum on the activity of respiratory lactate bypass might represent interest for strain engineering. However, for use of non-growing Ndh-deficient *Z. mobilis* cells as biocatalysts for aerobic bioethanol production, the lactate bypass is clearly an undesired side activity.
