# Supplementary material for: Aerobic catabolism and respiratory lactate bypass in Ndh-negative Zymomonas mobilis
Source: Metab Eng Commun. 2018 Nov 15;7:e00081. doi: 10.1016/j.mec.2018.e00081 (PMC6260413; doi:10.1016/j.mec.2018.e00081)
Supplement: Supplementary file 1 — Supplementary material [file mmc1.docx]

**Supplement Table1**

Growth parameters of aerobic and anaerobic batch cultures.

Aerobic cultivation was done in 200 mL shaken flasks with 20 mL culture, on a shaker at 180 r.p.m. Anaerobic cultivation was done in 18 mL glass screw cap tubes containing 10 mL of culture, which were vigorously gassed with nitrogen for a period of several minutes after inoculation

|  | Standard growth medium | | | | Medium supplemented with 30 mM D-lactate | | | |
| --- | --- | --- | --- | --- | --- | --- | --- | --- |
|  | Yx/glucose  [g dry wt mol^-1^] | | μ [h^-1^] | | Yx/glucose  [g dry wt mol^-1^] | | μ [h^-1^] | |
|  | aerobic | anaerobic | aerobic | anaerobic | aerobic | anaerobic | aerobic | anaerobic |
| *ndh-* | 7.77 ± 0.21 | 9.41 ± 0.41 | 0.36 ± 0.04 | 0.45 ± 0.05 | 9.36 ± 0.86 | 9.71 ± 0.27 | 0.37 ± 0.03 | 0.44 ± 0.06 |
| *ndh-ldh-* | 7.09 ± 0.36 | 8.01 ± 0.30 | 0.34 ± 0.01 | 0.42 ± 0.04 | 7.29 ± 0.41 | 8.10 ± 0.21 | 0.36 ± 0.03 | 0.42 ± 0.04 |
| *ndh-ldh+* | 8.48 ± 0.65 | 9.72 ± 0.54 | 0.38 ± 0.01 | 0.44 ± 0.02 | 9.31 ± 0.55 | 9.33 ± 0.33 | 0.36 ± 0.03 | 0.47 ± 0.03 |
